# A New Method for the Discovery of Essential Proteins

**DOI:** 10.1371/journal.pone.0058763

**Published:** 2013-03-21

**Authors:** Xue Zhang, Jin Xu, Wang-xin Xiao

**Affiliations:** 1 Key Laboratory of High Confidence Software Technologies, Ministry of Education, Peking University, Beijing, China; 2 School of Electronics Engineering and Computer Science, Peking University, Beijing, China; 3 Research Institute of Highway Ministry of Transport, Beijing, China; University of South Florida College of Medicine, United States of America

## Abstract

**Background:**

Experimental methods for the identification of essential proteins are always costly, time-consuming, and laborious. It is a challenging task to find protein essentiality only through experiments. With the development of high throughput technologies, a vast amount of protein-protein interactions are available, which enable the identification of essential proteins from the network level. Many computational methods for such task have been proposed based on the topological properties of protein-protein interaction (PPI) networks. However, the currently available PPI networks for each species are not complete, i.e. false negatives, and very noisy, i.e. high false positives, network topology-based centrality measures are often very sensitive to such noise. Therefore, exploring robust methods for identifying essential proteins would be of great value.

**Method:**

In this paper, a new essential protein discovery method, named CoEWC (Co-Expression Weighted by Clustering coefficient), has been proposed. CoEWC is based on the integration of the topological properties of PPI network and the co-expression of interacting proteins. The aim of CoEWC is to capture the common features of essential proteins in both date hubs and party hubs. The performance of CoEWC is validated based on the PPI network of *Saccharomyces cerevisiae*. Experimental results show that CoEWC significantly outperforms the classical centrality measures, and that it also outperforms PeC, a newly proposed essential protein discovery method which outperforms 15 other centrality measures on the PPI network of *Saccharomyces cerevisiae*. Especially, when predicting no more than 500 proteins, even more than 50% improvements are obtained by CoEWC over degree centrality (DC), a better centrality measure for identifying protein essentiality.

**Conclusions:**

We demonstrate that more robust essential protein discovery method can be developed by integrating the topological properties of PPI network and the co-expression of interacting proteins. The proposed centrality measure, CoEWC, is effective for the discovery of essential proteins.

## Introduction

Genome-wide gene deletion studies show that a small fraction of genes in a genome are indispensable to the survival or reproduction of an organism [1], [2]. These genes are referred as essential genes, and essential proteins are just the products of essential genes. The deletion of such essential proteins will result in lethality or infertility. The identification of essential proteins is very important not only for understanding the minimal requirements for survival of an organism, but also for finding human disease genes [3] and new drug targets. The genome-wide identification of essential genes is valuable for rational drug design [4]. Essential proteins in pathogenic organisms can be taken as the potential targets for new antibiotics [5].

Several experimental methods for the discovery of essential proteins have been conducted, such as single gene knockouts [6], RNA interference [7] and conditional knockouts [8]. However, these experimental methods are very time-consuming and laborious, and they often require large amounts of resources.

With the advances of high-throughput experimental technologies, such as Y2H and mass spectrometry, large amounts of protein-protein interaction (PPI) data have been produced, which make it possible to study proteins in network level. In order to break through experimental constraints, recently researchers have been paid more attention to computational methods based on network topological characteristics. The correlations between network topological features and protein essentiality have been explored by many researchers. It has been observed in several species, such as Saccharomyces cerevisiae, Caenorhabditis elegans and Drosophila melanogaster [9], [10], that hub proteins in PPI network, which highly connecting with other proteins, are more likely to be essential than those of low connections [11]. This is the so-called *centrality-lethality* rule [11]. Several researchers have tried to explain such correlation from different hypotheses [12]–[15]. Although some controversies exist among these explanations, most researchers have confirmed the correlation between topological centrality and protein essentiality [10], [16]–[18].

Computational methods could be seen as useful preprocessing techniques which could help experimental methods to quickly find essential proteins. Many centrality measures have been proposed to capture the correlation between network topological properties and protein essentiality. Local network features based centrality measures include degree centrality (DC) [11],sum of edge clustering (SoECC) [18], local average connectivity (LAC) [19], and density of maximum neighborhood component (DMNC) [20]. Global network characteristics based centrality measures include betweenness centrality (BC) [21], and closeness centrality (CC) [22]. Other previously proposed centrality measures include subgraph centrality [23], eigenvector centrality [24], information centrality [25], bottle neck [26], [27], and the method by integrating network topology and gene expression data (PeC) [28]. Comparative studies on the two kinds of measures show that local features based measures are more effective for identifying essential proteins [28]–[29].

Since the currently available PPI networks for each species are not complete, i.e. false negatives, and very noisy, i.e. high false positives, especially for those obtained by high-throughput technologies, the identification of essential proteins based on network topology is still very challenging. Most centrality measures are sensitive to such noise of PPI network. In addition, it is well known that both false negatives and false positives in PPI networks are hard to be cleaned out. Therefore, robust centrality measures for the discovery of essential proteins would be of great value. Biological information has been integrated with network topology to improve the precision of essential protein discovery methods [28], [30]. In [28], the authors proposed PeC method by integrating edge clustering coefficient and gene co-expression. In [30], essential proteins were explored based on the integration of network topological features and two types of GO annotations: cellular localization and biological process.

As reported in [13], essential proteins tend to form highly connected clusters rather than function independently. Some researchers began to pay attention to the relationship between protein essentiality and their cluster property [18], [31]. According to [32], hubs in the yeast interactome network can be classified into date and party hubs on the basis of their partners' expression profiles. This distinction suggests a model of organized modularity for the yeast proteome. Modules are connected through the date hubs which act as regulators, mediators or adaptors, while party hubs represent integral elements within the modules and tend to function at a lower level of the organization of proteome. That is, party hubs are well co-clustered with their neighbors in PPI network while date hubs are not. In addition, party hubs and date hubs have the similar probability to be essential [32]. Cluster-based centrality measures, such as clustering coefficient and sum of edge clustering coefficient, would be not effective for identifying essential proteins from date hubs.

With respect to these various difficulties and progresses, we propose a new centrality measure, named CoEWC, by integrating PPI data and gene expression data. CoEWC determines a protein's essentiality based on whether it has a high probability to be co-expressed with its neighbors and whether each of its neighbors takes part in densely connected clusters. Different from SoECC and PeC, which all emphasize co-clustering relationship between a protein and its neighbors, CoEWC pay more attention to the clustering property of the protein's neighbors rather than the protein itself. As we know, proteins within a cluster tend to share some similar biological functions with its neighbors and proteins with similar functions tend to be co-expressed. Therefore, we think that the co-expression of a protein with its interacting neighbors in PPI network can capture the co-clustering relationship between the protein and its neighbors to some extent. Moreover, CoEWC takes clustering properties of a protein's neighbors into consideration. As a result, CoEWC is expected to identify essential proteins from date hubs and party hubs well. The performance of CoEWC was tested on the well studied species of *Saccharomyces cerevisiae*. Compared to several previous centrality measures which have better predicting precision, CoEWC achieves higher predicting precision for the identification of essential proteins. The experimental results demonstrate that centrality measures, which based on the appropriate integration of network topological properties and gene expression, are more robust and effective, than those only based on network topological features, for the discovery of essential proteins, and that CoEWC is a good example for such integration.

## Methods

### Motivations

As reported in [32], hubs in the yeast interactome network can be classified into date and party hubs on the basis of their partners' expression profiles, and moreover, party hubs and date hubs have the similar probability to be essential. Therefore, exploring the co-expression between a protein and its interacting neighbors in PPI network to identify the protein's essentiality is reasonable.

If we use Pearson Correlation Coefficient (PCC) to capture the co-expression, we found in yeast interactome that some non-essential hubs tend to co-express with their neighbors with PCC values in a very large range from negative to positive. We take the protein YJR091C as an example to illustrate the phenomenon.

**Figure 1:**
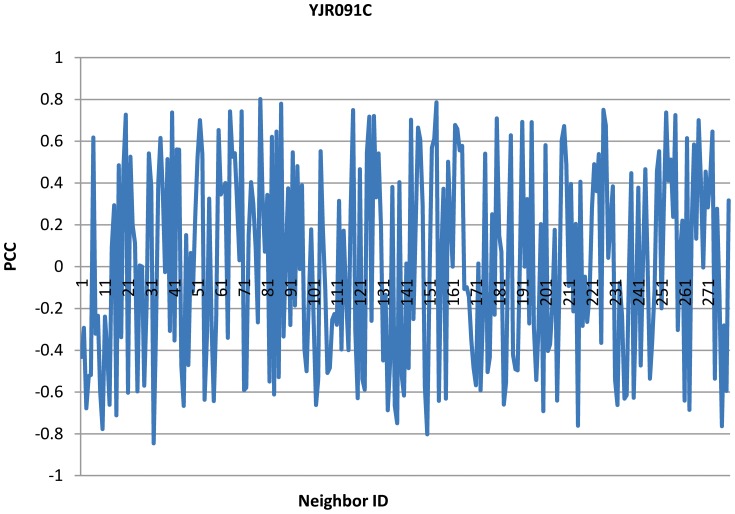
PCCs of YJR091C with its neighbors.

YJR091C is a non-essential hub protein in yeast proteome. It has the maximal degree, 280, in the yeast PPI network. YJR091C ranges the first according to DC and SoECC mainly due to its large degree. Now let us see its co-expression with its neighbors. Figure 1 shows the pearson correlation coefficients of YJR091C with its 280 neighbors. The PCC values ranges from −0.846 to 0.802. The sum of the PCC values is about 3.37, and YJR091C gets 451^th^ place according to sum of PCC. This tells us that PCC is more suitable to discriminate such non-essential proteins like YJR091C than DC and SoECC.

**Figure 2:**
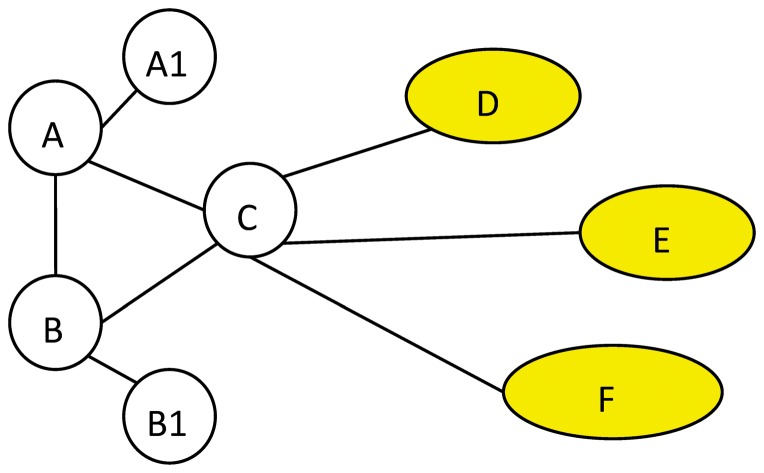
A toy network. A, A1, B, B1 and C are nodes of the toy network. D, E, and F are three complete sub-networks with size 20, 30, and 40. Node C connects with one node of D, E, and F respectively, say D1, E1 and F1.

Another motivation of the proposed centrality measure, CoEWC, can be demonstrated from the toy network in figure 2. Since edge clustering coefficient (ECC) measures whether two interacting nodes have a high probability to be co-clustered, according to the definition of SoECC [18], [28], edges AC and BC put more weight on determining node C's essentiality, than edges CD1, CE1 and CF1. However, this goes against our intuition. By intuition, edges CD1, CE1 and CF1 should put more weight on determining node C's essentiality. That is, on the basis of co-expression, it would be reasonable to take the clustering properties of a node's neighbors into consideration rather than the clustering property of the node itself.

By further observing the topological properties of date hubs and party hubs, we can know that essential proteins in these two kinds of hubs have very different clustering property themselves, but their neighbors tend to be of some common features, i.e. clustering property. Moreover, it is cheerful that such clustering property can also discriminate non-essential hubs to some extent. Centrality measures based on this idea will be more effective to find essential proteins from date hubs than those based on ECC. Clustering coefficient (CC) measures how well a node's neighbors are connected with each other, thus it can be used to capture a node's clustering property. According to CC, edges CD1, CE1 and CF1 put more weight on determining node C's essentiality than edges AC and BC in the toy network.

### New centrality measure: CoEWC

In this paper, a new centrality measure, CoEWC, is proposed based on the integration of PPI network and gene expression data. The basic ideas behind CoEWC are as follows: (1) A highly connected protein is more likely to be essential than a low connected one; (2) Essential proteins tend to form densely connected clusters; (3) Essential Proteins in the same cluster have a more chance to be co-expressed; (4) Party hubs and date hubs have the similar probability to be essential while they have very different clustering property. In CoEWC, a protein's essentiality is determined by the number of the protein's neighbors and the probability that the protein is co-expressed with its neighbors as well as its neighbors' clustering properties.

To describe the method simply and clearly, we give the following definitions and descriptions. The PPI network is represented by an undirected graph *G*(*V*, *E*), where a node *v*∈*V* represents a protein and an edge *e*(*u,v*)∈*E* denotes an interaction between two proteins *u* and *v*. Gene expression is the process by which information from a gene is used in the synthesis of a functional gene product. We only consider the gene expressions for proteins while some functional RNAs from non-protein coding genes may exist. For a protein *u*, its gene expressions with *s* different times are denoted as *Ge*(*u*) = {*g*(*u*,1),*g*(*u*,2),…,*g*(*u,s*)}. The probability that two proteins are co-expressed is evaluated based on the pearson correlation coefficient (PCC). The clustering property of a protein is evaluated based on the clustering coefficient (CC).

#### Pearson correlation coefficient

Pearson correlation coefficient (PCC) is a measure of the correlation between two variables, giving a value between +1 and -1 inclusive. It is widely used in the sciences as a measure of the strength of linear dependence between two variables. The PCC of a pair of genes (*X* and *Y*), which encode the corresponding paired proteins (*u* and *v*) interacting in the PPI network, is defined as:
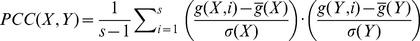
(1)


Where *s* is the number of samples of the gene expression data; g(*X*,*i*) (or g(*Y*,*i*)) is the expression level of gene *X* (or *Y*) in the sample *i* under a specific condition; 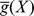 (or 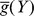) represents the mean expression level of gene *X* (or *Y*) and 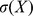 (or 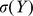) represents the standard deviation of expression level of gene *X* (or *Y*).

The pearson correlation coefficient of a pair of proteins (*u* and *v*) is defined as the same as the PCC of their corresponding paired genes (*X* and *Y*), that is *PCC*(*u,v*) = *PCC*(*X,Y*). If *PCC*(*u,v*) has a positive value, there is a positive linear correlation between *u* and *v*.

As we know, co-clustered proteins tend to share some similar functions and proteins with similar functions tend to be co-expressed. That is, two proteins *u* and *v* with a larger value of *PCC*(*u,v*) are more likely to be in the same cluster and to function similarly.

#### Clustering coefficient

In graph theory, a clustering coefficient is a measure of degree to which nodes in a graph tend to cluster together. Evidence suggests that in most real-world networks, and in particular social network, create tightly knit groups characterized by a relatively high density of ties [33], [34]. Yeast PPI network is also a small world network. Two versions of this measure exist: the global and the local. The global version was designed to give an overall indication of the clustering in the network, whereas the local gives an indication of the embeddedness of single nodes. Here, we refer to the local clustering coefficient.

The local clustering coefficient of a node in a graph quantifies how close its neighbors are to being a clique (complete graph). Watts and Strogatz introduced the measure in 1998 to determine whether a graph is a small world network [34]. The local clustering coefficient for a protein *u* in PPI network can be defined as
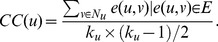
(2)


Where *N_u_* is the set of neighbors of protein *u* and *k_u_* denotes the number of immediately connected neighbors of *u*.

*CC*(*u*) is a local variable which characterizes the clustering property of a protein *u*. A protein *u* with a larger value of *CC*(*u*) is expected to put more impact on its neighbors, which has demonstrated in section 2.

#### CoEWC method

It has been proved that there exist a number of protein complexes which play a key role in carrying out biological functionality [35] and essential proteins tend to form protein complexes [36]. In addition, essential proteins in the same cluster tend to be co-expressed. It seems that centrality measures by exploring the co-clustering and co-expression properties for a protein will work well for the task of identifying essential proteins, just like PeC does. PeC outperforms many previous centrality measures indeed. However, as reported in [32], hubs can be divided into date hubs and party hubs, and these two kinds of hubs tend to be essential with similar probability. PeC mainly emphasizes the co-clustering and co-expression properties of a protein with its neighbors, so it would be not effective to identify essential proteins from date hubs which are not well co-clustered with its neighbors. According to the analysis on a toy network in the section of motivations, SoECC may capture the wrong features for date hubs, which make it not effective for identifying essential proteins from date hubs.

Although date hubs and party hubs have very different co-clustering property, each of their neighbors may have the similar co-clustering property. For a party hub, each of its neighbors is generally also a member of the same densely connected module that the hub involves in. So by exploring the clustering property of each of the hub's neighbors, we can capture the hub's high degree property. For date hubs, they often mediate different densely connected modules. Generally each neighbor of a date hub involves in a densely connected cluster, though the clusters its neighbors involve in are often different. By exploring each of its neighbors' own clustering property, we can also capture the high degree property of a date hub, and filter hubs whose neighbors are seldom connected with other proteins. Hubs with large number of disconnected neighbors tend to be non-essential.

In order to capture the characteristics of essential proteins based on the above standpoints, we propose a new centrality measure which is named as CoEWC. We use PCC to capture the co-clustering and co-expression properties of a protein with its neighbors, and use local clustering coefficient to capture the high connectivity of a protein and also each of its neighbors' clustering property.

For a protein *u*, its *CoEWC*(*u*) is defined as the sum of the PCC between *u* and each of its neighbors weighted by the corresponding neighbor's clustering coefficient. The definition is given in equation (3).
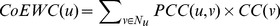
(3)


Where *N_u_* denotes the set of all immediately connected neighbors of node *u* in PPI network.

From the above analysis and the definition of CoEWC, CoEWC can identify essential proteins from both party hubs and date hubs, and can discriminate those non-essential hubs whose neighbors are mainly disconnected single proteins.

## Results and Discussion

### Test data

To evaluate the performance of the proposed new centrality measure, CoEWC, the PPI network and gene expression data of *Saccharomyces cerevisiae* was used, as it has been well characterized by knockout experiments and widely used in the evaluation of methods for essential proteins discovery. The test data used in this paper come from [28]. We describe them briefly as follows.

The PPI data of *Saccharomyces cerevisiae* was downloaded from DIP database [37]. There are 24,743 interactions among 5093 proteins in total after the self-interactions and the repeated interactions were filtered. The PPI network consists of 21 components. The largest component consists of 5052 proteins.

Essential proteins of *Saccharomyces cerevisiae* were collected from several databases, such as MIPS, SGD, DEG and SGDP. Out of all the 5093 proteins in the PPI network, 1167 proteins are essential among which 1165 proteins are in the largest component of the PPI network.

The gene expression data of *Saccharomyces cerevisiae* was retrieved from [38], containing 6,777 gene products and 36 samples in total. There are 4,981 proteins have the corresponding gene expression data while other 112 proteins have no corresponding gene expression data among which 6 proteins are essential. For proteins which have no corresponding gene expression data, we simply set them with zero values.

### Compare CoEWC with other centrality measures

In order to validate the performance of the proposed new centrality measure, CoEWC, we carry out a comparison between it and several state-of-the-art centrality measures: Degree Centrality (DC) [11], Sum of Edge Clustering Coefficient (SoECC) [18], PeC [28] and Clustering Coefficient (CC) [34].

The reasons that we choose these four centrality measures to compare are as follows. DC has been proved to be a good indicator for protein essentiality by many researchers [11], [28], and by comparing with it, we want to show the ability of CoEWC to identify essential proteins from hub proteins. SoECC is a better method for the discovery of essential proteins among the centrality measures only based on network topological property [18], [28]. PeC is a centrality measure also based on the integration of PPI data and gene expression data and outperforms 15 previous centrality measures in yeast PPI network: Betweenness Centrality (BC), Closeness Centrality, Subgraph Centrality (SC), Eigenvector Centrality (EC), Information Centrality (IC), Bottle Neck (BN), Density of Maximum Neighborhood Component (DMNC), Local Average Connectivity-based method (LAC), Sum of ECC (SoECC), Range-Limited Centrality (RL), L-index (LI), Leader Rank (LR), Normalized α-Centrality (NC), and Moduland-Centrality (MC). Therefore, we only compare CoEWC with PeC, but don’t compare with many mainstream centrality measures outperformed by PeC in the yeast PPI network for identifying essential proteins. PeC aims to capture the co-clustering property of a protein with its neighbors from both a topological view and a biological view. However, CoEWC aims to capture the properties of both date hubs and party hubs while the two hubs have very different clustering property. CC is used to show how many improvements can be obtained by properly integrating it with gene expression data, just like CoEWC does.

**Figure 3:**
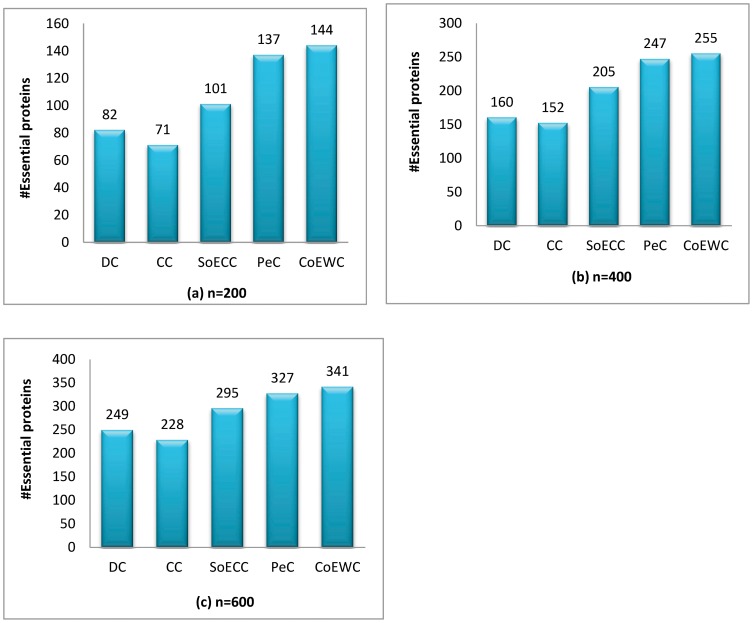
Comparison of the number of essential proteins detected by CoEWC and other four previously proposed centrality measures.

Figure 3 gives the comparison of the number of essential proteins detected by CoEWC and other four previously proposed centrality measures. Proteins are ranked according to their values calculated by each centrality measure. For each centrality measure, a certain number of top proteins are selected as candidates for essential proteins, out of which the number of true essential proteins is determined.

From figure 3 we can see that CoEWC significantly outperforms the centrality measures only based on network topological features (DC, CC and SoECC) for predicting essential proteins from yeast PPI network. CoEWC also outperforms PeC for predicting more essential proteins than PeC does. Especially, CoEWC obtains more than 50% improvement over DC and CC for predicting 500 proteins, and obtains about 20% improvement over SoECC. There is more than 4% improvement of CoEWC over PeC for predicting 600 proteins.

### Validated by jackknife methodology

**Figure 4:**
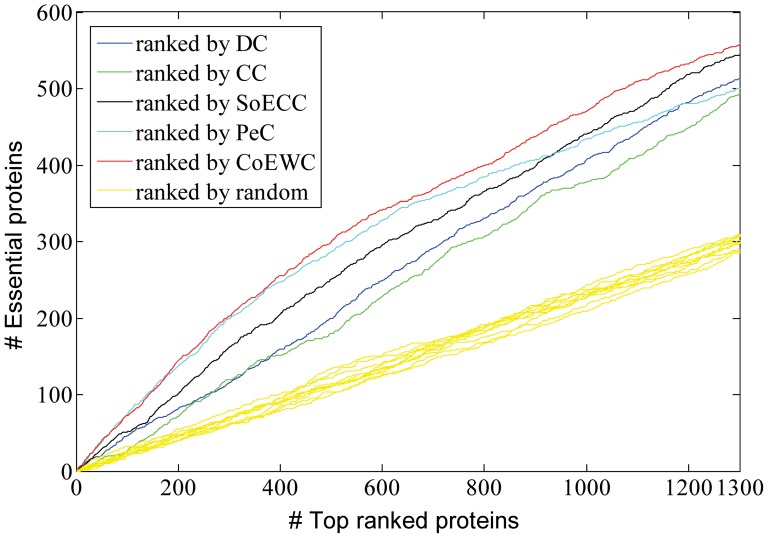
Comparison results by a jackknife methodology.

Now we use jackknife methodology [39] to test the comparison between the proposed centrality measure CoEWC and other four previously proposed centrality measures (DC, CC, SoECC and PeC). The comparison results are shown in figure 4. In figure 4, proteins are ordered from the highest value to the lowest value for each centrality measure and the cumulative counts of essential proteins are plotted. The areas under the curve (AUC) for CoEWC and other centrality measures are compared. In addition, ten random assortments are also plotted for comparison.

As shown in figure 4, it is clear that the sorted curve of CoEWC appears to be much better than three centrality measures: DC, CC and SoECC. For top 180 ranked proteins, CoEWC ties with PeC. Then the sorted curve of CoEWC is increasingly better than that of PeC with the increase of the number of top ranked proteins. All the results of the five centrality measures are better than those of randomized sortings. In figure 4, the AUC of CoEWC is 4.3174e+005, and the AUC of PeC is 4.0450e+005. This tells us that CoEWC is more effective than PeC for the task of identifying essential proteins. Therefore, our idea that capturing the properties of both date hubs and party hubs by using the co-expression of a protein with its neighbors weighted by the corresponding neighbor's clustering coefficient is better than that only capturing the co-clustering property of a protein with its neighbors.

### Analysis of the differences between CoEWC and the compared centrality measures

To further analyze why and how CoEWC performs well on the identification of essential proteins, we study the relationship and difference between it and other compared centrality measures (DC, CC, SoECC, and PeC) by predicting a small fraction of proteins. The top 200 proteins are selected for each centrality measure.

Firstly, we compare CoEWC with the other four centrality measures (DC, CC, SoECC and PeC) by investigating how many proteins are both predicted by CoEWC and by anyone of the other four centrality measures. The number of overlaps between CoEWC and one of the other centrality measures is shown in table 1. |*CoEWC* ∩ *M_i_*| denotes the number of common proteins detected by CoEWC and by a centrality measure *M_i_*. {*M_i_ - CoEWC*} (or {*CoEWC -M_i_*}) denotes the set of proteins identified by *M_i_* (or *CoEWC*) not by CoEWC or (*M_i_*), and |*M_i_ - CoEWC*| is the number of proteins identified by *M_i_* not by CoEWC.

**Table 1:** The relationships between CoEWC and four centrality measures for predicting the top 200 proteins.

Centrality measures (*M_i_*)	|*CoEWC* ∩ *M_i_*|	|*M_i_ - CoEWC*|	Non-essential proteins in {*M_i_ -CoEWC*}	Non-essential proteins in {*CoEWC - M_i_* }	Percentage of non-essential proteins in {*M_i_ - CoEWC*} with low CoEWC
Degree Centrality (DC)	60	140	95	33	49.5%
Clustering Coefficient (CC)	19	181	123	50	69.9%
Sum of ECC (SoECC)	78	122	70	27	52.9%
PeC	155	45	19	12	21.1%

From table 1, we can see that the common proteins identified by CoEWC and DC, CC are not more than 30%, that common proteins predicted by CoEWC and SoECC are less than 40%, and that common proteins both predicted by CoEWC and PeC are less than 80%. The small overlap between the predicted proteins of CoEWC and DC, CC shows that CoEWC is a special centrality measure which is much different from classical centrality measures. In addition, we investigated the non-essential proteins predicted by other centrality measures, and found that about 50% of these non-essential proteins predicted by three network topology-based centrality measures (DC, CC and SoECC) are with very low values of CoEWC (less than 0.128) and there are 21.1% of the non-essential proteins predicted by PeC are with very low values of CoEWC (less than 0.128).

**Figure 5:**
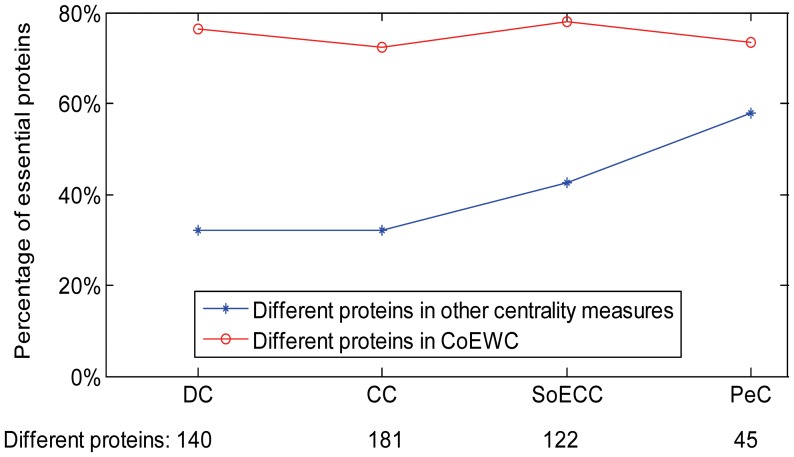
Comparison of the percentage of essential proteins out of all the different proteins between CoEWC and other four centrality measures: DC, CC, SoECC and PeC.

Secondly, we evaluate the different proteins identified by CoEWC and those by other centrality measures. Figure 5 gives the number of proteins which are predicted out of all the different proteins identified by CoEWC and those identified by DC, CC, SoECC and PeC. As shown in figure 5, the percentage of essential proteins identified by CoEWC is consistently higher than that identified by each other centrality measures for the different proteins between them. Take CC as an example, which has the largest different number of proteins from CoEWC. Out of all the top 200 proteins, 181 proteins are differently identified by CC and by CoEWC, respectively. Out of these 181 proteins of CoEWC, 72.4% proteins are essential, while only 32% proteins out of the 181 proteins by CC are essential. Then take PeC as an example, which has the smallest difference from CoEWC. There are 45 different proteins identified by CoEWC and by PeC. Out of 45 different proteins, CoEWC identified more than 73.3% essential proteins while PeC only identified less than 57.8% essential proteins. The similar results can be obtained from the DC and SoECC.

There are 26 proteins which are predicted by CoEWC but not included in any of the top 200 proteins of the other four centrality measures. These 26 proteins are shown in table 2. In table 2, we show the following information of the 26 proteins: the rank in CoEWC, protein name, degree, value in CoEWC and essentiality. As shown in table 2, out of the 26 proteins 80.8% are essential.

**Table 2:** List of 26 proteins predicted by CoEWC but are ignored by other four centrality measures DC, CC, SoECC, and PeC when predicting the top 200 proteins.

Rank	Protein Name	Degree	CoEWC	Essentiality
104	YDR365C	23	1.698583	essential
124	YDL232W	18	1.503577	essential
127	YJL033W	19	1.49437	essential
128	YGL099W	14	1.492625	essential
130	YBR234C	23	1.473234	essential
131	YIL075C	32	1.472131	essential
139	YLR200W	10	1.435959	non-essential
145	YDL087C	23	1.401966	essential
147	YKL095W	38	1.390057	essential
151	YHR081W	5	1.358778	non-essential
152	YPR088C	14	1.351337	essential
154	YOL094C	37	1.327599	essential
156	YHL030W	21	1.310346	non-essential
158	YOR259C	28	1.282556	essential
161	YBL041W	9	1.275247	essential
163	YNL182C	21	1.268987	essential
170	YMR314W	16	1.246819	essential
178	YBR126C	29	1.219773	non-essential
179	YOL142W	6	1.219508	essential
181	YBL023C	14	1.211368	essential
187	YNL290W	34	1.176549	essential
190	YFL008W	24	1.156859	essential
191	YPL012W	20	1.153688	essential
193	YER025W	26	1.140787	essential
194	YOR210W	16	1.138906	essential
199	YKL068W	35	1.119936	non-essential

**Figure 6:**
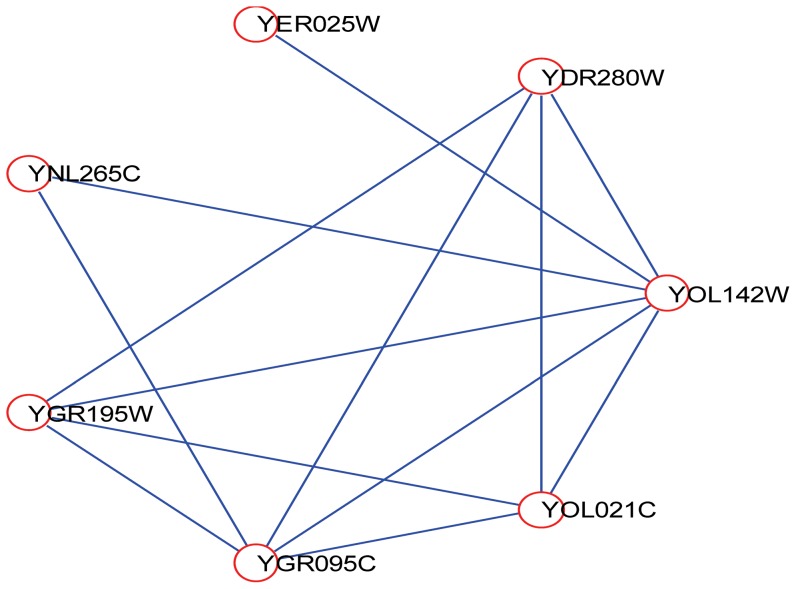
YOL142W and its interacting neighbors.

Take YOL142W as an example. YOL142W is an essential protein whose degree is only 6. The interactions between YOL142W and its neighbors are shown in figure 6. To further study the characteristic of YOL142W and its neighbors, we show the following information of its neighbors: PCC value, CC value, and essentiality in table 3. From table 3, we can see that its 5 neighbors out of all 6 neighbors are also essential proteins, and that YOL142W is well co-expressed with its 5 neighbors which are also essential. All the CC values of its neighbors are significantly larger than the average CC value of the whole PPI network which is 0.097. Table 3 also tells us that co-clustered essential proteins tend to be co-expressed and that CoEWC can capture this property well.

**Table 3:** Information of the neighbors of YOL142W.

Proteins	PCC	CC	Essentiality
YDR280W	0.8046	0.3399	essential
YER025W	0.639	0.1354	essential
YNL265C	−0.357	0.1648	non-essential
YGR195W	0.771	0.4083	essential
YGR095C	0.7414	0.3897	essential
YOL021C	0.8391	0.375	essential

Take another non-essential protein, YLR295C, as an example. YLR295C has 125 neighbors, out of which only 24 are essential. YLR295C gets its rank of 16, 2388, and 8 according to DC, CC, and SoECC, respectively. According to the definition of DC, CC and SoECC and the corresponding ranks of YLR295C according to these three centrality measures, we can conclude that YLR295C is a hub protein and is well co-clustered with some of its neighbors, and that there are very few connections between its neighbors (its CC value is only 0.0017). It is obvious that YLR295C cannot be discriminated by DC and SoECC.

**Figure 7:**
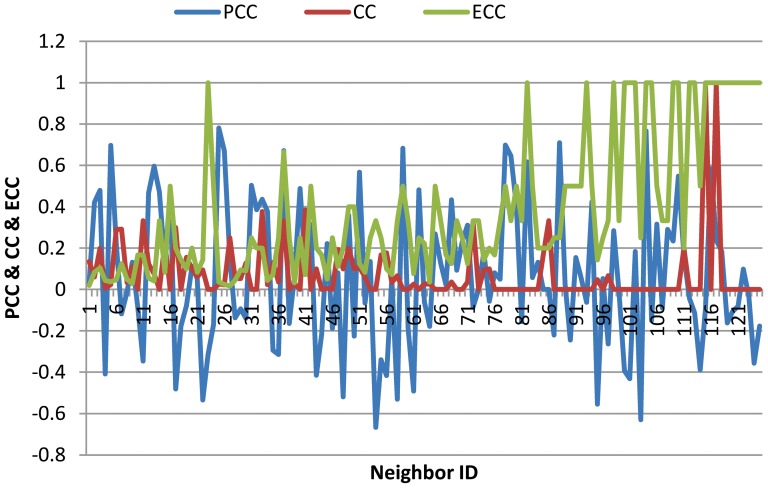
Properties of 125 neighbors of YLR295C.

In addition, in order to further compare CoEWC with PeC, we also compute the sum of PCC (SoPCC) between a protein and all its neighbors in PPI network, and rank all proteins according to SoPCC. YLR295C gets its rank of 121 according to SoPCC and gets the rank of 123 according to PeC. Figure 7 gives the properties of YLR295C and its 125 neighbors captured by PCC, CC and ECC. In figure 7, the neighbor proteins with first 24 neighbor IDs are essential, and the other proteins are non-essential.

From the distributions of PCC, CC and ECC in figure 7, we can see that about a quarter of its neighbors are well co-clustered with YLR295C (with ECC value equals to 1), but only very few neighbors have large CC values. Almost all ECC values of the neighbors are larger than zero, but about half of the neighbors' CC values are zero. Among YLR295C's interacting proteins, essential proteins tend to have non-zero CC values, which accords with the assumption that essential proteins tend to be co-clustered with some of its neighbors. According to the definition of SoECC, which is the sum of ECC, YLR295C's SoECC value is large due to its high degree. According to the definition of PeC, which is the sum of the product of ECC and PCC, the PeC value of YLR295C is considerably smaller than that of SoECC due to the negative values of PCC. Moreover, according to the definition of CoEWC, which is the sum of the product of PCC and CC, the CoEWC value of YLR295C is smaller than those of SoECC and PeC, due to both negative PCC values and smaller CC values. From figure 7, we can further understand why CoEWC can discriminate YLR295C as non-essential while DC, SoECC and PeC cannot.

Table 4 shows a list of non-essential proteins which have a high degree but with a low value of CoEWC. In order to compare with other centrality measures, we also give their values of DC, CC, SoECC and PeC. Since the values predicted by different centrality measures are not comparable directly, here we take the 1110^th^ proteins' values, which are sorted in descending order according to each centrality measure, as reference values. The reference values for DC, CoEWC, CC, SoECC and PeC are 12, 0.127, 0.1428, 3.057 and 0.55, respectively. From table 4, we can see that these non-essential proteins cannot be discriminated by DC and SoECC. By considering the co-expression properties between a protein and its interacting proteins, both CoEWC and PeC have an improved discrimination ability over these non-essential proteins. As shown in table 4, all these non-essential proteins with a high degree consistently have a very low value of CoEWC. From the above analysis, we can see that CoEWC can help filter the false predictions of other centrality measures. So the integration of PCC and CC is effective for the prediction of essential proteins.

**Table 4:** List of non-essential proteins which have a high degree but with a low value of CoEWC.

Protein Name	Essentiality	DC	CoEWC	CC	SoECC	PeC
YCL018W	non-essential	156	0.0723	0.0244	21.2481	0.1766
YBR127C	non-essential	113	0.0502	0.0207	11.7541	−0.2884
YMR106C	non-essential	110	-0.2571	0.0283	18.9553	−0.543
YLR288C	non-essential	99	−0.1364	0.0033	31.4151	3.3742
YLR191W	non-essential	97	−1.5164	0.0215	24.9253	−1.6514
YLR447C	non-essential	95	−0.0429	0.0035	26.2248	2.4992
YOL055C	non-essential	93	0.0942	0.0140	8.1786	−0.3599
YHR135C	non-essential	84	−0.2160	0.0203	13.849	−0.3505
YGR040W	non-essential	79	0.1058	0.0207	8.6171	0.3374
YLR453C	non-essential	78	0.0411	0.003	23.4248	0.3608
YER118C	non-essential	72	−0.2675	0.0274	14.6054	−0.2627
YDL059C	non-essential	67	0.1163	0.0407	7.3506	−0.0661
YCL027W	non-essential	67	0.0730	0.009	18.7385	4.1962
YBL085W	non-essential	67	−0.2364	0.0212	13.4245	−0.8018
YGR254W	non-essential	67	−0.0167	0.0298	7.08	−0.0244
YAR014C	non-essential	65	−0.20841	0.016346	13.29514	−2.5911
YDR171W	non-essential	61	−0.28158	0.023497	4.637564	0.0205
YHR140W	non-essential	60	−0.55414	0.249718	30.04586	−2.13
YML048W	non-essential	60	−0.44267	0.136723	18.25437	−0.4479
YGR262C	non-essential	60	−0.09723	0.029379	7.759587	0.4257
YJL098W	non-essential	59	−0.26835	0.04851	10.58723	−0.5065
YLR096W	non-essential	58	−0.27887	0.047792	11.02443	−0.6211
YJL095W	non-essential	58	−0.03866	0.050817	13.54987	0.7098
YGL237C	non-essential	57	−0.39525	0.025063	10.00474	0.8943
YNL135C	non-essential	57	0.046016	0.022556	8.115793	0.1006
YDR386W	non-essential	57	−0.65712	0.030702	6.746308	−1.4038
YCL040W	non-essential	55	−0.11312	0.020875	5.221536	0.0757
YDL101C	non-essential	55	0.069512	0.041077	9.862956	−0.4066
YGL173C	non-essential	52	−0.80869	0.032428	5.137779	−0.8367
YER179W	non-essential	50	−0.14783	0.04	6.687021	−0.357
YKL065C	non-essential	50	−0.3737	0.173061	15.15515	−0.7295

## Conclusions

With the large amount of PPI data available for some species, the discovery of essential proteins from network level is becoming a hot topic. Many network topology-based centrality measures for the discovery of essential proteins have been proposed. However, the currently available PPI networks for each species are incomplete (false negatives) and very noisy (high false positives). At the same time, most of the network topology-based methods depend on the reliability of the available protein-protein interactions and thus are very sensitive to the network. Moreover, essential proteins may be of distinct clustering properties, i.e. date hubs and party hubs, at the same time essential and non-essential proteins are often of some common features, i.e. high degree for hub proteins. It is very challenging to well capture the true distinct features for essential proteins to distinguish them from non-essential proteins.

To tackle the above difficulties, we propose a new centrality measure, named CoEWC, based on the integration of PPI data and gene expression data. CoEWC aims to capture the common features of essential proteins in both date hubs and party hubs by integrating PCC with CC together. CoEWC is applied to the PPI network of *Saccharomyces cerevisiae*. The experimental results show that CoEWC significantly outperforms the network topology-based centrality measures: DC, CC and SoECC, and that CoEWC also outperforms PeC, a currently proposed centrality measure which also based on the integration of PPI data and gene expression data.

Although CoEWC performs well on the discovery of essential proteins, there should be still a space to improve the prediction precision. First, the integration of PCC and CC is very simple in this paper, and there may exist more abstruse relationship between PCC and CC. Second, there should exist some more excellent method to well capture the distinct properties between essential proteins and non-essential proteins. Finally, besides the gene expression data, some other protein related data, such as biological process, domain information, and localization, should be also valuable for the task of identifying essential proteins.
